# Molecular Mechanisms of Programmed Cell Death During Somatic Embryo Detachment from Callus in Indirect Somatic Embryogenesis

**DOI:** 10.3390/ijms27188421

**Published:** 2026-09-21

**Authors:** Haibing Huang, Zhongzheng Ma, Tiantian Sun, Fude Wang

**Affiliations:** 1 Heilongjiang Institute of Wood Science, Harbin 150081, China; 2 State Key Laboratory of Tree Genetics and Breeding, College of Forestry, Northeast Forestry University, Harbin 150040, China

**Keywords:** vacuolar autolysis, execution proteases, cell wall remodeling, tissue abscission, LAFL transcription factors, in vitro regeneration

## Abstract

Somatic embryogenesis in plants occurs via direct or indirect routes. In indirect somatic embryogenesis, embryos initiate from callus. The detachment of these embryos from callus is a critical step for plantlet recovery. Most reviews focus only on embryo induction. This review focuses specifically on the detachment stage. We examine how programmed cell death (PCD) drives this physical release. Cell death occurs in boundary callus cells and the suspensor. It does not destroy the developing embryo proper. PCD performs three main tasks. It dissolves middle lamella pectins. It removes the temporary suspensor. And it digests doomed cells to recycle nutrients for the embryo. Key proteases such as metacaspases execute this death program, with vacuolar processing enzymes representing candidate execution proteases. Epigenetic marks and transcription factors restrict cell death to the boundary. We also discuss practical ways to regulate PCD in tissue culture.

## 1. Introduction

Somatic embryogenesis is a unique process. Somatic cells switch fate and form complete plants without gamete fusion [1,2]. This process occurs only in plants. It provides a major system for clonal propagation, genetic transformation, and germplasm preservation [2,3]. In tissue culture, this process follows two distinct routes [4]. The first route is direct somatic embryogenesis (DSE) [5]. In DSE, embryos form directly on explant surfaces without an intermediate callus phase [5,6]. The explant feeds the developing embryo through a suspensor [6]. The second route is indirect somatic embryogenesis (ISE) [7,8]. In ISE, explants first dedifferentiate into unorganized callus [8,9]. Embryogenic cells then differentiate within this callus and develop into bipolar embryos [9,10].

In ISE, somatic embryos must detach from the callus matrix to mature [10,11]. Incomplete detachment leads to fused embryos and poor conversion [12,13]. Early research focused on cell wall changes and hormone shifts during separation [14,15]. Recent studies show that programmed cell death (PCD) drives this physical detachment [16,17]. PCD is a genetically regulated cell suicide program [18]. It shapes plant organs, removes temporary structures, and aids nutrient recycling [19,20]. In embryogenic callus, PCD targets specific boundary cells [17,21]. This localized cell death creates the physical gap needed for embryo release [21,22].

Previous reviews summarized general embryogenesis pathways [23,24]. Others covered zygotic embryogenesis [25] or general plant cell death [26,27]. Yet none synthesized the specific mechanisms that detach somatic embryos from callus. Key questions remain open. We do not fully understand which proteases execute boundary cell death. We do not know how transcription factors restrict death to the interface. And we lack data on how PCD coordinates with cell wall loosening. This narrative review addresses these gaps. We evaluate the cytological, enzymatic, and molecular cascades that control somatic embryo detachment in indirect somatic embryogenesis.

## 2. Cell Biological Characteristics and Molecular Regulation of Somatic Embryogenesis

### 2.1. Process and Stage Characteristics of Somatic Embryogenesis

Somatic embryogenesis is a multi-step developmental process that can be divided into three main stages: induction, differentiation, and maturation [10]. During the induction stage, explants undergo dedifferentiation under appropriate hormone and culture conditions to form callus; during the differentiation stage, some cells within the callus acquire embryogenic characteristics, forming embryogenic cell clusters; during the maturation stage, the embryogenic cell clusters further develop into somatic embryos with a complete bipolar structure [10,11].

In plant tissue culture, somatic embryogenesis follows two distinct routes: direct (DSE) and indirect (ISE) [4,5]. In DSE, embryos arise directly from explant tissues without an intervening callus phase, and their detachment relies on suspensor PCD [6]. In ISE, explants first dedifferentiate into callus, from which embryogenic cell clusters redifferentiate and must detach through the dismantling of boundary callus parenchyma [7,8]. This review focuses on the detachment mechanisms in ISE, while drawing functional comparisons with suspensor PCD in DSE and zygotic systems [28,29].

The development of somatic embryos follows a pattern similar to zygotic embryos. They pass through globular, heart-shaped, torpedo-shaped, and cotyledonary stages [12]. The process is complete when mature embryos germinate and convert into intact plantlets. In indirect somatic embryogenesis, embryos must detach from the callus before they can germinate normally. In terms of cell morphology, embryogenic cells exhibit significant differences from non-embryogenic cells. Embryogenic callus is typically soft, white or cream-colored, composed of isodiametric cells with dense cytoplasm, prominent nuclei, and very small vacuoles, and mitotic activity is observed [13]. In contrast, non-embryogenic callus is harder, yellow to brown in color, with large, highly vacuolated cells and thick cell walls, and almost no cell division activity is observed [14]. At the ultrastructural level, embryogenic cells contain typical eukaryotic cytoplasmic components, including nuclei, mitochondria, and vacuoles, with abundant ribosomes in the cytoplasm, indicating active metabolic activity [14].

### 2.2. Molecular Differentiation Between Embryogenic Cells and Callus Cells

Embryogenic cells differ from non-embryogenic callus cells in gene expression [15]. In upland cotton, embryogenic cells upregulate *LEC1*, *WOX5*, *ABI3*, *FUS3*, and *WRKY2* [16]. Similar transcriptional shifts occur in other species. In *Arabidopsis*, BBM, *LEC2*, and AGL15 direct embryogenic reprogramming [17,19]. In carrot, somatic embryogenesis requires the SERK1 receptor kinase and DC8 expression [30,31]. In Norway spruce, *PaWOX8/9* and *PaVP1* direct proembryogenic mass differentiation [32,33]. These shared transcription factors establish embryogenic cell identity across angiosperms and gymnosperms. Further studies have shown that the formation of embryogenic cells involves cell cycle reactivation and cell fate reprogramming [17]. During the transition from somatic cells to embryogenic cells, cells must undergo dedifferentiation, activation of the cell division cycle, and reorganization of physiological, metabolic, and gene expression patterns [18]. This process is regulated by multiple factors, including stress, endogenous growth regulators, and chromatin remodeling. In particular, the interaction between stress and hormone signaling pathways plays an important role in the induction of somatic embryogenesis.

### 2.3. Molecular Regulatory Network of Somatic Embryogenesis

Studies in *Arabidopsis* divide somatic embryogenesis regulation into five distinct levels [19]. Level 1 is the developmental state and embryogenic competence of the explant tissue. Level 2 is auxin signaling, which alters chromatin accessibility across the genome. Level 3 comprises master transcription factors, including BBM, *LEC1*, and *LEC2* [20,21]. Level 4 consists of downstream embryonic gene networks, including *FUS3* and *ABI3* [17,22]. Level 5 comprises epigenetic regulators that stabilize embryogenic cell identity. These five tiers work together during somatic cell reprogramming.

Epigenetic mechanisms control this cell fate switch [23]. DNA hypomethylation at SERK and WUS promoter loci promotes embryogenic induction [34]. Histone acetylation by *HAG1* relaxes chromatin packaging at embryogenic genes [35]. In contrast, the histone deacetylase complex factor PKL represses embryonic traits during vegetative growth [36]. MicroRNAs also direct this transition [23]. miR396 regulates GRF transcription factors to control cell division [20]. *miR160* and *miR167* target *ARF* genes to modulate auxin sensitivity [37].

## 3. Functional Mechanisms of Programmed Cell Death (PCD) in Plant Embryogenesis

van Doorn classified plant programmed cell death into autolytic and non-autolytic types [24]. Autolytic PCD features tonoplast rupture and rapid cytoplasmic clearance [24]. Non-autolytic PCD lacks rapid protoplast clearance and occurs during pathogen defense [24]. During plant embryogenesis, vacuolar-type autolytic PCD represents the predominant morphological mode [25]. Cells undergoing autolytic PCD display progressive cytoplasmic vacuolation, organelle degradation, and nuclear condensation [26]. Tonoplast collapse occurs at the terminal stage, discharging vacuolar processing enzymes and hydrolases that clear the protoplast and leave behind a modified cell-wall corpse [25]. In somatic embryogenesis systems, this tightly regulated autolytic process is deployed during both suspensor elimination and embryo–callus boundary remodeling.

## 4. Specific Mechanisms of PCD During Somatic Embryo Detachment from Callus

### 4.1. PCD-Mediated Detachment Mechanism of Embryogenic Cell Clusters

**Figure 1:**
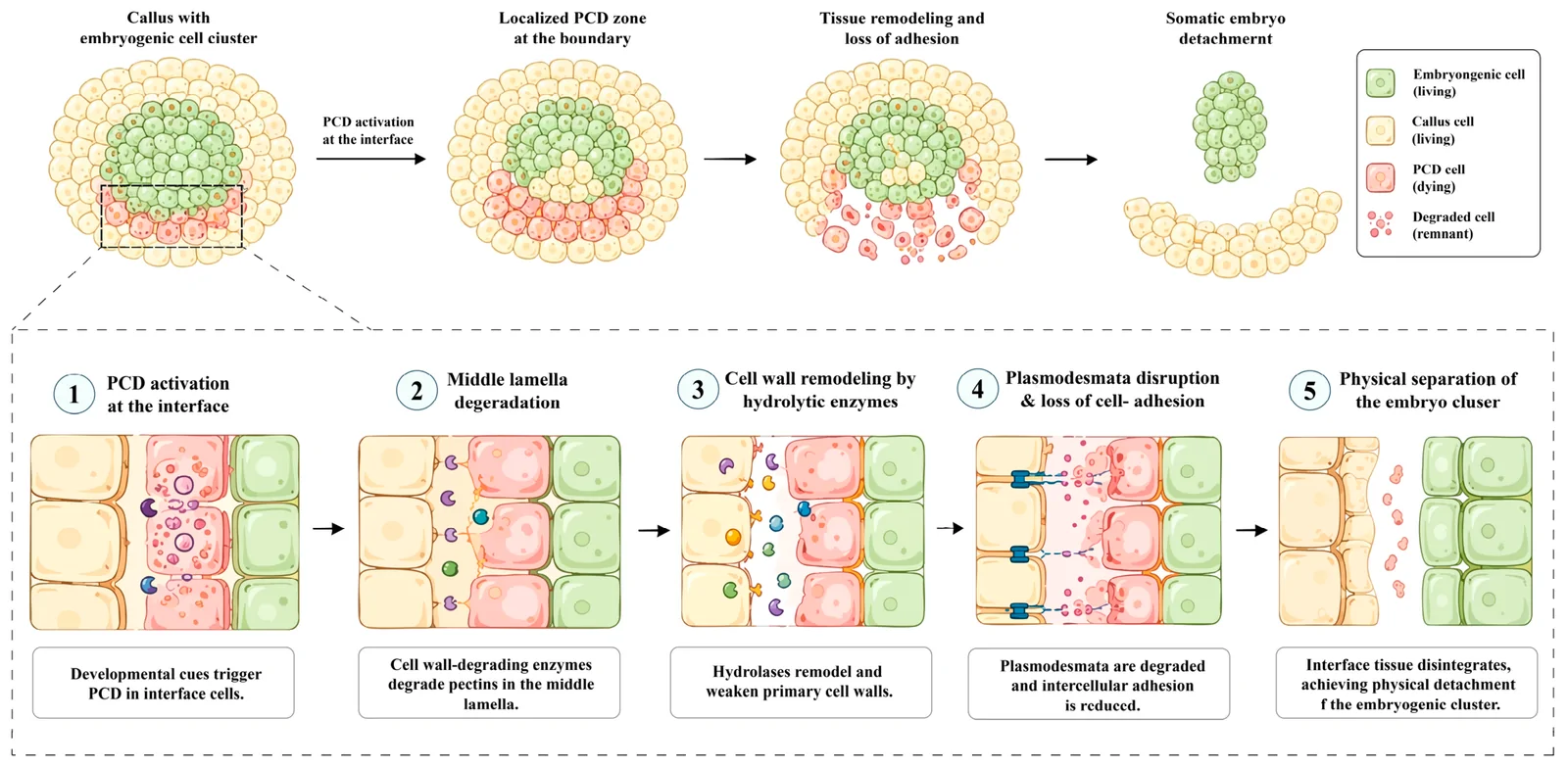
Proposed conceptual model of somatic embryo detachment from callus. Boundary cells undergo cell death. Pectinases digest the middle lamella. Cell walls loosen and plasmodesmata break. The embryo then detaches from the callus. Note that boundary cell death was shown in cotton and carrot. But the exact step-by-step wall breakdown is inferred from other plant abscission systems.

**Figure 2:**
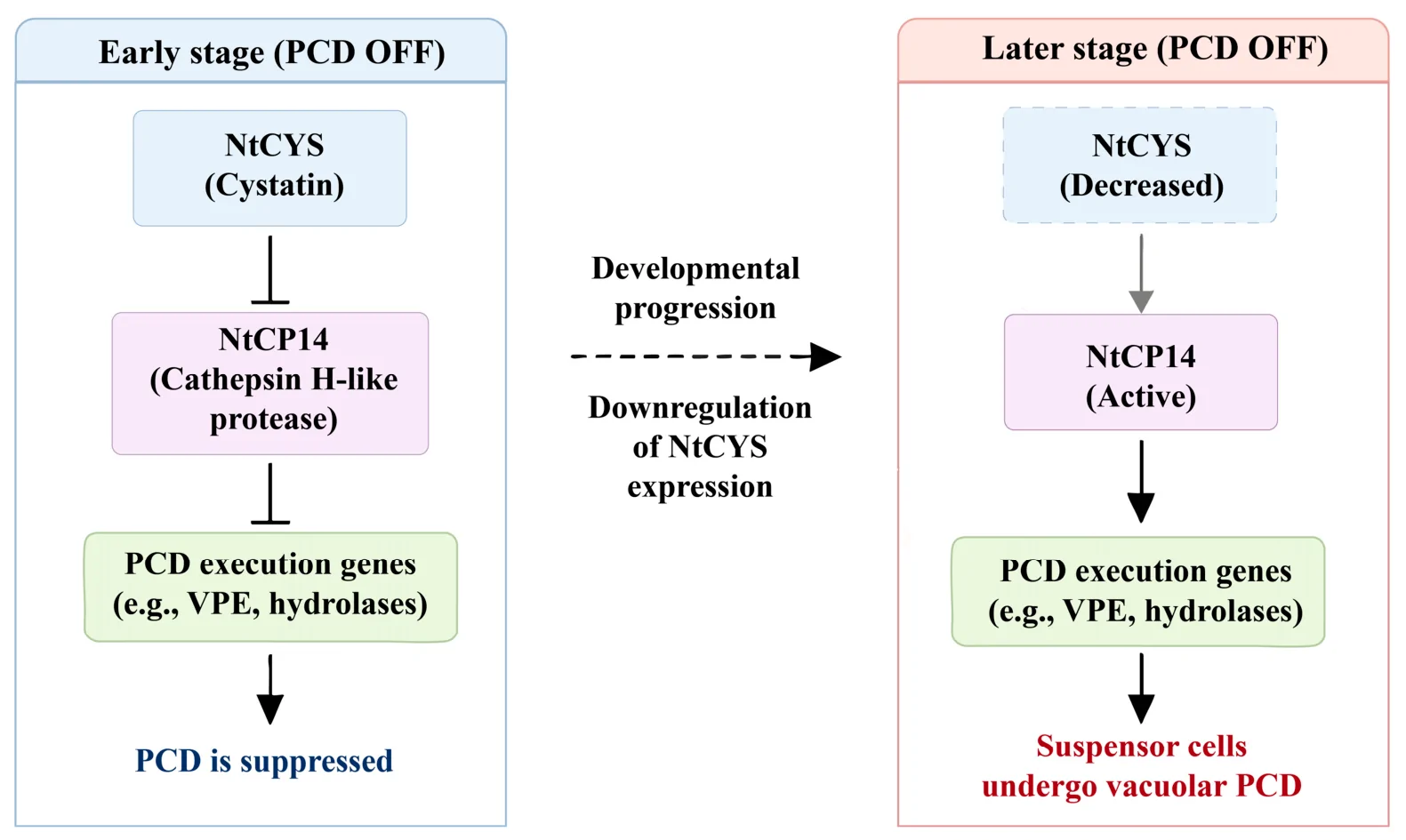
The *NtCYS*-*NtCP14* inhibitory switch in suspensor cell death. In early embryos, *NtCYS* binds *NtCP14* and blocks cell death. Later, *NtCYS* levels decrease. Active *NtCP14* then triggers vacuolar cell death in suspensor cells. This switch serves as a comparative genetic model for boundary-restricted cell death.

Unlike direct embryogenesis, where detachment relies solely on suspensor breakdown, indirect somatic embryogenesis faces an additional hurdle: the embryogenic cluster is physically embedded in callus tissue. Its release requires localized programmed cell death in the boundary parenchyma cells that connect the cluster to the surrounding callus. This is not merely a passive separation but a regulated process. Histological data from wheat [12] suggest that early-stage clusters are still linked to callus via plasmodesmata and intact middle lamellae, whereas mature, detached embryos appear only after the connecting cells have been eliminated, likely through PCD. In cotton, a PCD peak around day 10 of culture coincides with the appearance of DNA laddering and nuclear condensation specifically at the cluster–callus interface [35]. Similar observations in carrot suspension cultures indicate that vacuolization and cell death in the centre of embryogenic clusters weaken internal cohesion, allowing peripheral meristematic cells to separate as new embryos [37]. Thus, boundary PCD serves as the physical release mechanism unique to the indirect pathway [38,39]. Figure 1 illustrates this five-step detachment process at the callus boundary. First, developmental cues trigger PCD in interface cells (Step 1). Second, dying cells release pectinases that degrade middle lamella pectins (Step 2). Third, hydrolases loosen primary cell walls (Step 3). Fourth, plasmodesmata break down, reducing cell-to-cell adhesion (Step 4). Fifth, the embryogenic cluster physically separates from the callus (Step 5). We do not depict a suspensor in Figure 1. Figure 1 shows the broad parenchyma boundary detachment in indirect embryogenesis. Suspensor PCD occurs in direct embryogenesis and is shown separately in Figure 2.

### 4.2. PCD Eliminates Redundant Structures During Somatic Embryo Development

Suspensor elimination occurs in both zygotic and somatic embryos through conserved pathways [11,40]. In both systems, the suspensor is a temporary structure. It anchors the early embryo and channels nutrients [11]. In zygotic embryos, suspensor cells die via vacuolar PCD as the embryo enters the heart-shaped stage [39]. Somatic embryos in conifers and specific dicots form suspensors that die at the same stage [41,42]. Both embryo types use vacuolar autolysis to clear suspensor cells [42,43]. But somatic embryos on callus also require boundary parenchyma death for release. In tobacco, an *NtCYS*-*NtCP14* protein switch triggers suspensor PCD (Figure 2) [42]. *NtCYS* inhibits the protease *NtCP14* in early embryos. When *NtCYS* levels decrease, active *NtCP14* initiates suspensor cell death [42]. This switch shows how cells restrict death to specific boundaries. In Horse Chestnut (*Aesculus hippocastanum* L.) [41] and *Arabidopsis* [42], around the heart-shaped stage of somatic embryo development, suspensor cells gradually undergo vacuolar cell death and are eliminated through vacuolar PCD. The molecular mechanism of suspensor PCD involves a complex regulatory network. The mechanism triggering suspensor PCD is based on the antagonistic action of two proteins: the protease inhibitor cystatin *NtCYS* and its target, the cathepsin H-like protease *NtCP14* [43]. *NtCYS* is expressed in the basal cell of the proembryo, and the encoded cystatin binds to *NtCP14* and inhibits its activity, thereby preventing premature PCD. The anti-cell death effect of *NtCYS* is subject to transcriptional regulation and is suppressed by the 32-cell embryo stage, leading to increased *NtCP14* activity and initiation of PCD (Figure 2).

### 4.3. PCD Participates in Nutrient Redistribution for Somatic Embryos

PCD also plays an important role in nutrient redistribution during somatic embryo development [43]. Research indicates that during vacuolar cell death, the lytic vacuole gradually engulfs and digests the cytoplasmic contents [44]. This process not only eliminates unwanted cellular structures but also releases nutrients from the cellular contents for use by surrounding living cells. This nutrient recycling and redistribution mechanism is of great significance for the normal development of somatic embryos, particularly under in vitro culture conditions where external nutrient supply is limited, making the efficient utilization of endogenous nutrients especially important [44].

PCD-mediated tissue remodeling and nutrient recycling are intimately coupled with cellular antioxidant dynamics and redox homeostasis [45]. Comparative studies between embryogenic callus and degenerating embryogenic callus have found that the concentrations of proteins, reduced glutathione (GSH), and ascorbic acid (ASC), as well as the ratio of ASC to dehydroascorbic acid (DHA), are significantly higher in embryogenic callus than in degenerating embryogenic callus [45]. These antioxidants not only protect embryogenic cells from oxidative damage but may also participate in the stabilization and transport of nutrients during PCD.

**Figure 3:**
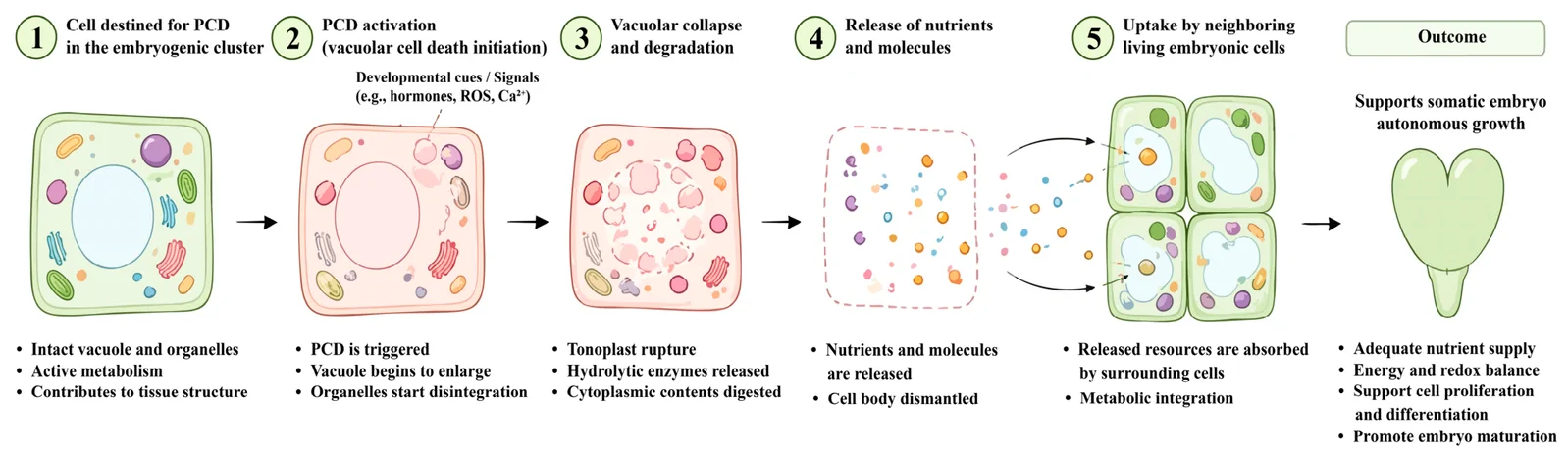
Proposed conceptual model of nutrient redistribution during somatic embryogenesis. Vacuoles digest cellular contents. Dying cells release metabolites. Neighboring cells take up these resources. This sequence is a proposed model based on transporter expression and autolysis data.

PCD also recycles cellular materials during embryo maturation [43,46]. As boundary cells die, lytic vacuoles digest proteins, nucleic acids, and lipids [44,46]. This autolysis releases free amino acids, peptides, and sugars into the apoplast [44,45]. Surviving embryogenic cells absorb these metabolites to sustain growth [47,48]. In Norway spruce, senescing suspensor cells upregulate specific peptide transporters and amino acid permeases [49]. This transporter activation supports active nutrient remobilization. Figure 3 illustrates this five-step recycling sequence: (1) death initiation in boundary cells; (2) vacuole enlargement; (3) tonoplast rupture and cytoplasmic digestion; (4) release of nutrients and metabolites; and (5) nutrient uptake by neighboring living embryonic cells.

## 5. Molecular Regulatory Network and Signal Transduction Mechanisms of PCD

### 5.1. Functional Mechanisms of Key PCD Execution Proteins

The execution of PCD in plants involves multiple key proteins, among which metacaspases are the most important class. During plant embryogenesis, metacaspase *mcII-Pa* is translocated from the cytoplasm to the nucleus of terminally differentiated cells destined for elimination, where it co-localizes with the nuclear pore complex and chromatin, causing nuclear envelope disintegration and DNA fragmentation. The cell death function of *mcII-Pa* depends on its cysteine-dependent arginine-specific proteolytic activity, and mutation of the catalytic cysteine eliminates the proteolytic activity of *mcII-Pa* and blocks nuclear degradation.

Metacaspases exhibit developmental specificity in their role in plant PCD. In situ hybridization analysis shows that *mcII-Pa* mRNA accumulates restrictively in doomed embryonic tissues and structures. In early embryos, transcript accumulation occurs in the suspensor, with no hybridization signal detected in the embryogenic mass composed of proliferating cells that will further differentiate into the mature embryo. In mature embryos, *mcII-Pa* expression is associated with procambial strands, representing an early stage of xylem differentiation [48].

Vacuolar processing enzyme (*VPE*) is another important class of PCD execution proteins. *VPE*s possess YVADase, caspase-1-like substrate specificity, and have caspase-like catalytic dyads, catalytic sites, and aspartate substrate pockets. *VPE* activity is upregulated following *Tobacco mosaic virus* (TMV) infection, with the active enzyme localizing to the vacuole, regulating the maturation of vacuolar proteins during senescence and stress-induced cell death [50]. *VPE* cleaves substrates with caspase-1-like specificity. It mediates vacuolar collapse in plant tissues. But researchers have not tested *VPE* during embryo detachment from callus. Its role in this process remains a hypothesis.

Caspase-like activities increase during embryo development [51]. Plants lack animal caspases. Instead, other plant proteases cleave these substrates [50]. For example, VEIDase activity rises during early embryogenesis [51,52]. Blocking VEIDase stops normal suspensor development [52].

### 5.2. Transcription Factor Network Regulating PCD

Somatic embryogenesis and programmed cell death are governed by distinct yet coordinated transcriptional modules. These four transcription factors—LEC1, ABI3, FUS3, and LEC2—are collectively referred to as the LAFL group. While core transcription factors such as *LEC1*, *LEC2*, *FUS3*, *ABI3*, and *RKD4* primarily establish embryogenic cell identity and drive embryo proper morphogenesis [53], they concurrently delineate the boundary between viable embryonic tissues and doomed structures destined for PCD [54]. Rather than acting as ubiquitous cell death triggers, these embryonic master regulators modulate hormonal gradients (notably auxin biosynthesis via YUC genes) and chromatin accessibility, thereby creating the developmental context required for localized PCD execution. In contrast, the direct triggering and spatial restriction of PCD in suspensor and boundary cells are executed by specialized downstream modules—such as cystatin–cathepsin protease complexes and metacaspases—ensuring that cell dismantling remains strictly confined to the abscission zone without compromising embryo proper viability [43,48].

**Figure 4:**
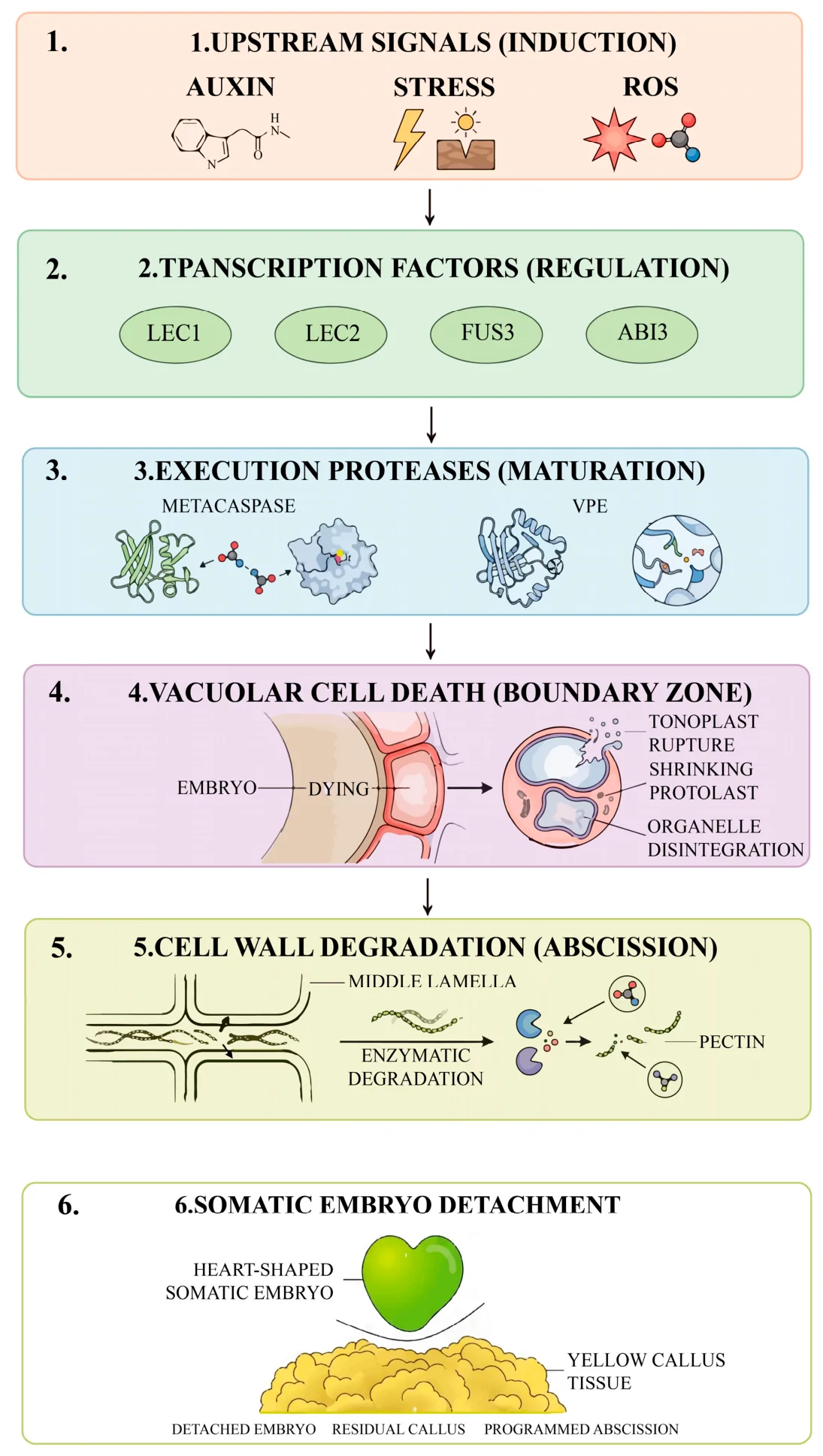
Sequential pathway of somatic embryo detachment. Master regulators set tissue boundaries. Cells activate execution proteases and undergo cell death. The middle lamella dissolves. The embryo then detaches from the callus. Solid arrows show tested steps.

*LEC1* (LEAFY COTYLEDON1) belongs to the HAP3 transcription factor family and, together with LEAFY COTYLEDON1 LIKE (*L1L*), is a key regulator of zygotic embryogenesis. Ectopic overexpression of *LEC1* leads to early seedling growth arrest and abnormal plant development, occasionally accompanied by the formation of somatic embryo-like structures, thus conferring somatic embryogenic potential [55]. During somatic embryogenesis, *LEC1* expression is subject to epigenetic regulation, with a hypomethylated state of its promoter region promoting its transcriptional activation [56]. *LEC2*, *FUS3*, and *ABI3* constitute another important transcription factor regulatory module [57]. The three transcription factors form a positive feedback loop essential for activating the embryogenesis program in somatic cells [58]. *LEC2* also increases the transcription of two flavin monooxygenases, *YUC2* and *YUC4*, responsible for auxin synthesis [1,57]. Post-embryonic ectopic expression of *LEC2* leads to the formation of somatic embryos, callus, and cotyledon/leaf-like structures, while downregulation of *LEC2*, *FUS3*, and *ABI3* significantly inhibits both direct and indirect somatic embryogenesis. RKD4 (RWP-RK domain 4) is a plant-specific transcription factor belonging to the RWP-RK transcription factor family. Ectopic expression of RKD4 induces the expression of embryogenesis-associated genes within 8 days, initiates embryogenesis in somatic cells, and promotes somatic embryogenesis, whereas constitutive ectopic expression of RKD4 leads to continuous proliferation without differentiation. RKD4 plays roles in regulating both suspensor and embryo body development, and the diversity of its expression patterns and functions makes it an important factor in PCD regulation during somatic embryogenesis [59]. Figure 4 illustrates this sequential signaling-to-execution pathway during embryo detachment. Upstream stress, auxin, and ROS signals activate master transcription factors (*LEC1*, *LEC2*, *FUS3*, *ABI3*) [19,57]. These regulators set developmental boundaries between the embryo proper and surrounding tissues [54]. Boundary cells then activate execution proteases, including metacaspases and *VPE*s [48,50]. The execution of PCD causes vacuolar rupture and cell death in boundary and suspensor cells [25]. Dying cells release pectinases and cellulases that dissolve the middle lamella [38,40]. This enzymatic wall breakdown releases the somatic embryo from the parent callus.

### 5.3. Epigenetic Regulatory Mechanisms

Epigenetic marks help guide somatic embryo detachment. Somatic cells dedifferentiate and change their transcription patterns over several weeks [18]. During this phase, stress and culture hormones modify chromatin [60]. These changes reset cell fate. Cells remodel chromatin packaging to switch from unorganized division to embryo development.

DNA methylation regulates gene transcription across the genome [61]. Enzymes add methyl groups to cytosine bases in CpG, CpHpG, and CpHpH contexts. Three methyltransferase families catalyze these transfers [62]. These enzymes include chromomethylases (CMT), domain-rearranged methyltransferases (DRM), and methyltransferases (MET) [62]. In Arabidopsis, DRM2 adds de novo marks, while DRM2, MET1, and CMT3 maintain methylation patterns during DNA replication [63]. Methylation patterns shift during somatic embryogenesis. The LEC1 promoter loses methyl groups before embryogenesis begins [64]. Methylation then increases as embryos mature and enter vegetative growth [64]. Targeting RNA-directed DNA methylation to the LEC1 promoter reduces its transcription [64]. Treating cells with 5-aza-2′-deoxycytidine blocks methyltransferases, boosting embryogenesis and raising STM transcription [64].

Histone modifications also direct cell reprogramming. Histone acetylation removes positive charges from basic histone tails. This reaction weakens histone binding to DNA, relaxing chromatin into an open state [65]. Open chromatin permits transcription of embryogenic genes [65]. Cells then switch from proliferation to organized embryo growth [65]. In Arabidopsis, the PICKLE gene encodes a CHD3 histone deacetylase subunit [66]. Knocking down PICKLE raises LEC1 transcription [66]. This knockdown delays the transition from embryonic to vegetative growth [66].

### 5.4. Signal Transduction Pathways and Regulatory Networks

Calcium ions transmit signals during programmed cell death. These signals travel to the nucleus and alter transcription. Calmodulin and domain kinases relay calcium-dependent inputs [67]. MAP kinases also phosphorylate target proteins without calcium [67]. Other transcription factors bind signal complexes directly [67]. Auxin pathways work alongside calcium [68]. LEC2 increases auxin synthesis genes and totipotency factors, including BBM and LEC1 [68]. This creates a feed-forward loop that reinforces cell fate [68]. Auxin directs cell division in embryonic tissues [19]. It also tunes PCD gene expression to help detach embryos [19].

**Figure 5:**
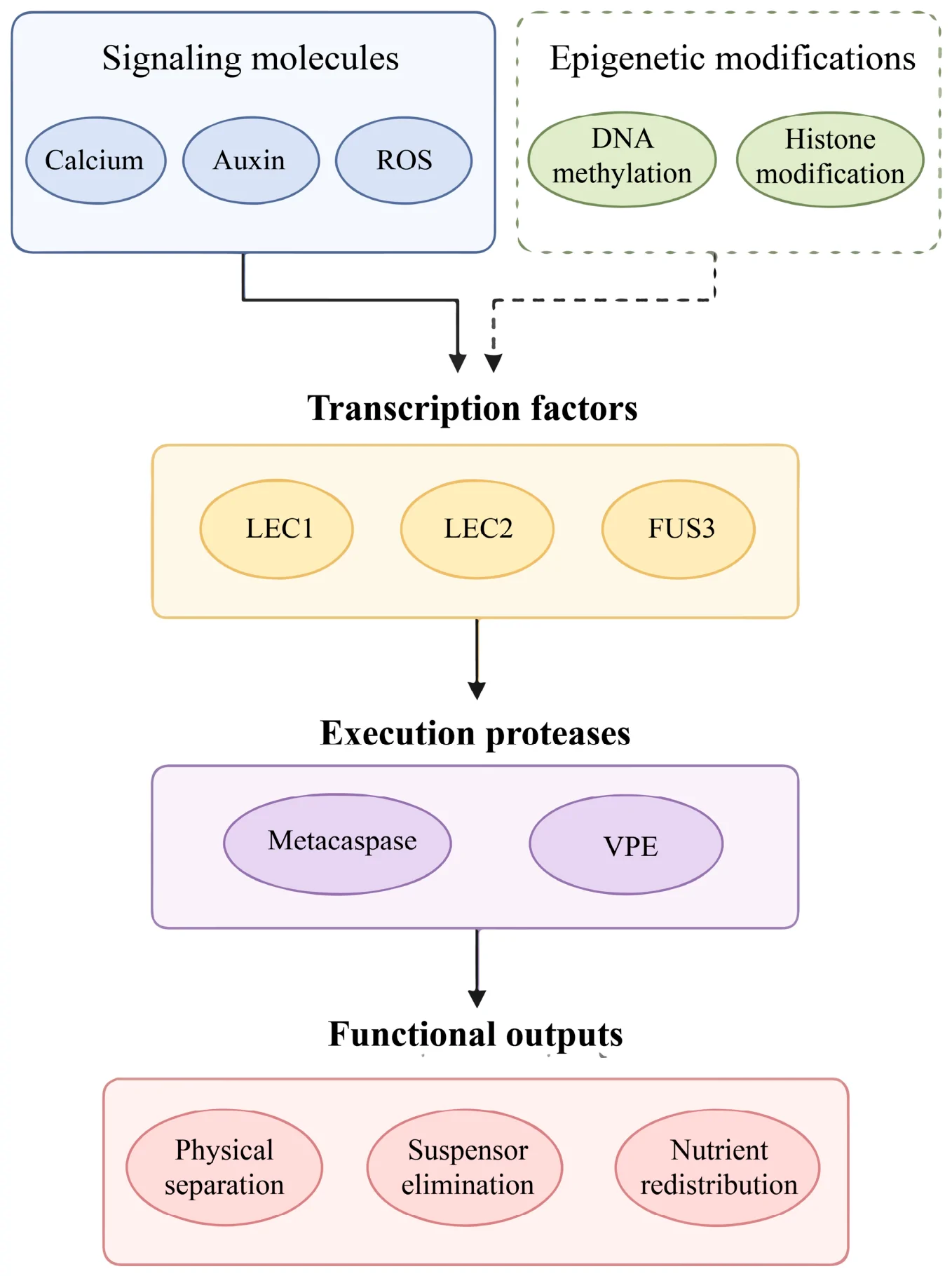
Molecular regulatory network in somatic embryo detachment. Signals, chromatin marks, and transcription factors interact during detachment. Solid lines show demonstrated links. Dashed lines show proposed cross-talk.

Reactive oxygen species act in a dose-dependent manner [47,69]. Low ROS levels maintain cell division and pluripotency. Embryogenic cells need these basal oxidative cues. But excess ROS kills cells [47]. Boundary cells produce oxidative bursts that damage lipids and membranes. This stress activates proteases and causes localized cell death. Antioxidant levels and ROS amounts decide cell fate. Researchers can adjust these signaling nodes to manage cell death. Controlling this balance improves embryo yield and quality in culture. Signals, transcription factors, chromatin marks, and execution proteases work together in a connected network (Figure 5).

## 6. Application Value of PCD in Somatic Embryo Culture Technology

### 6.1. Improving Somatic Embryo Induction Efficiency by Regulating PCD

Regulating PCD improves embryo yield and detachment in bioreactors [8,60]. Adjusting dissolved oxygen levels alters ROS production [61]. A controlled oxidative pulse triggers boundary PCD, accelerating embryo detachment. But excessive ROS damages embryonic tissues. Supplementing media with antioxidants such as ascorbic acid or glutathione protects the embryo proper while allowing boundary separation [45,62]. Temporary auxin withdrawal downregulates protease inhibitors like *NtCYS*, promoting suspensor and boundary PCD [42]. Once detached, transferring embryos to hormone-free media induces histodifferentiation and shoot–root meristem formation [63,64]. The experimental evidence for PCD components in different plant embryogenic systems is summarized in Table 1.

### 6.2. Industrial Applications of Somatic Embryo Culture Technology

Somatic embryo culture technology has broad application prospects in forest tree breeding and crop improvement [8]. Significant progress has been made in the application of somatic embryo culture technology in plants. Molecular analyses of signaling pathways and processes in conifer embryogenesis, such as programmed cell death and embryo maturation, indicate that many developmental pathways are conserved between angiosperms and gymnosperms [70]. This finding provides an important theoretical foundation for optimizing conifer somatic embryo culture technology.

Artificial seed technology is another important direction for the application of somatic embryos. Somatic embryos possess complete bipolar structures and can germinate into whole plants like seeds, making them ideal materials for producing artificial seeds [71]. By encapsulating somatic embryos in artificial seed coats and adding appropriate nutrients and protective agents, artificial seeds with germination capability can be produced [72]. This technology has important application value in the conservation of rare plants, rapid propagation of superior varieties, and preservation of germplasm resources. Detachment quality directly affects artificial seed success. Incomplete detachment leaves dead callus on the embryo base. This tissue blocks gas exchange inside alginate beads. It also increases microbial growth during storage. Clean detachment ensures healthy embryos that convert into plants after sowing.

### 6.3. Technological Innovations Based on PCD Mechanisms

Translating PCD mechanisms into tissue culture requires defined protocols [73,74]. Single-cell culture systems allow researchers to track early cell fate transitions without interference from surrounding callus tissues [73]. Applied plant cell biology methods show that fine-tuning microenvironmental factors alters boundary cell viability [74]. For example, temporary auxin withdrawal downregulates protease inhibitors, triggering localized PCD in boundary cells. Once embryos detach, transfer to hormone-free media promotes shoot–root meristem differentiation [66,67]. In bioreactors, real-time tracking with pH-sensitive fluorescent biosensors will allow growers to monitor boundary cell death dynamics during large-scale embryo production [75].

We divide current applications into two categories. Proven tissue culture methods adjust oxygen, light, and antioxidants [8,45]. These methods alter ROS levels and improve embryo yields in crops and trees [70]. In contrast, genetic approaches remain prospective. Using CRISPR to edit protease genes is still in development [73]. Real-time biosensors in bioreactors also require more testing [75]. This distinction separates practical culture protocols from future molecular tools.

## 7. Knowledge Gaps and Future Research Priorities

We identify five priority areas for future research:

1. Single-Cell and Spatial Transcriptomics: Current studies analyze bulk callus samples. This masks differences between individual cells. Single-cell RNA sequencing will map gene expression at the boundary.
2. Live-Cell Imaging: The timing of vacuolar collapse remains unknown in living tissue. Fluorescent biosensors will track pH, ROS, and protease activity during detachment.
3. Genetic Validation: Most proteases lack functional proof. Researchers must use CRISPR to knock out candidate genes and test their roles.
4. Cell-Wall Analysis: We do not know how proteases trigger pectin degradation. Future work must track cell-wall enzymes during PCD.
5. Evolutionary Comparisons: Somatic embryogenesis differs between gymnosperms and angiosperms. Comparative studies will reveal if detachment mechanisms are conserved across seed plants.

## 8. Conclusions

In indirect somatic embryogenesis, programmed cell death drives somatic embryo detachment from callus. PCD performs three main tasks: boundary separation, suspensor removal, and nutrient recycling. It is executed by metacaspases and cell wall hydrolases under transcription factor control, with VPEs representing candidate proteases that await functional validation in the detachment context. In plant tissue culture, embryo development is directed by controlled nutrient and hormone supplies rather than being fully autonomous. Clean detachment frees the embryo from parent callus, allowing it to absorb media nutrients directly and proceed through histodifferentiation. Applying single-cell transcriptomics, CRISPR gene editing, and live biosensors will resolve interface mechanisms and improve micropropagation protocols.

## Figures and Tables

**Table 1:** Summary of experimental evidence for PCD components in plant embryogenic systems.

Species & System	Developmental Stage	PCD Markers & Regulators	Proposed Function	Direct Callus Detachment?	Ref.
*Gossypium hirsutum* (Cotton, indirect SE)	Callus differentiation (Day 10)	TUNEL assay, DNA laddering ROS, Auxin	Boundary tissue remodeling and cluster release	Yes (Directly verified at callus–embryo interface)	[35]
*Daucus carota* (Carrot, indirect SE)	Globular cluster disintegration	Cell vacuolation, corpse clearance Extracellular hydrolases	Central cluster breakdown and embryo release	Yes (Observed during cluster separation)	[37]
*Picea abies* (Norway spruce, ESM)	PEM-to-somatic embryo transition	Nuclear fragmentation mcII-Pa, VEIDase activity	Removal of PEM cells and suspensor	Partial (Suspensor verified; ESM separation)	[25,33,48,52]
*Nicotiana tabacum* (Tobacco, zygotic/proembryo)	Globular to heart-shaped stage	Cell lysis, viability staining NtCYS–NtCP14 module	Suspensor PCD initiation and spatial restriction	No (Comparative model for suspensor PCD)	[42]
*Aesculus hippocastanum* (Horse chestnut, indirect SE)	Heart-shaped stage	Ultrastructural autolysis (TEM) Vacuolar hydrolases	Suspensor degradation during embryo maturation	No (Suspensor only, not callus boundary)	[39]
*Arabidopsis thaliana* (Direct/indirect SE)	Induction & globular embryo stage	Promoter-reporter assays LEC1, LEC2, FUS3, ABI3, RKD4	Embryonic fate determination and boundary setting	Indirect (TFs specify embryo identity vs. callus)	[17,19,57,59]

## Data Availability

No new data were created or analyzed in this study. Data sharing is not applicable to this article.
